# Biomimetic spectro-temporal features for music instrument recognition in isolated notes and solo phrases

**DOI:** 10.1186/s13636-015-0070-9

**Published:** 2015

**Authors:** Kailash Patil, Mounya Elhilali

**Affiliations:** Department of Electrical and Computer Engineering, Center for Speech and Language Processing, Johns Hopkins University, Baltimore, MD, USA

## Abstract

The identity of musical instruments is reflected in the acoustic attributes of musical notes played with them. Recently, it has been argued that these characteristics of musical identity (or timbre) can be best captured through an analysis that encompasses both time and frequency domains; with a focus on the modulations or changes in the signal in the spectrotemporal space. This representation mimics the spectrotemporal receptive field (STRF) analysis believed to underlie processing in the central mammalian auditory system, particularly at the level of primary auditory cortex. How well does this STRF representation capture timbral identity of musical instruments in continuous solo recordings remains unclear. The current work investigates the applicability of the STRF feature space for instrument recognition in solo musical phrases and explores best approaches to leveraging knowledge from isolated musical notes for instrument recognition in solo recordings. The study presents an approach for parsing solo performances into their individual note constituents and adapting back-end classifiers using support vector machines to achieve a generalization of instrument recognition to off-the-shelf, commercially available solo music.

## 1 Introduction

Research into the nature of musical timbre often focuses on the role of physical attributes of each musical instrument and how it colors the sound produced to give it its unique identity. The literature often enumerates spectral and temporal identifiers of musical timbre. Spectral information is historically the most studied dimension for musical instrument identification. It spans the magnitude spectrum envelope or relative amplitude of harmonic partials [1–3], number of harmonics [4], spectral centroid [5–7], spectral energy distribution [7, 8] and spectral irregularity [9]. Temporal characteristics of musical notes are equally important in shaping sound identity; including onset information [10], temporal envelope profile [11], energy buildup or attack over time [3, 12], vibrato [13, 14] as well as spectral flux over time [9]. Indeed, most studies have converged on the fact that a *joined* space of spectral and temporal information is necessary to fully describe the space of musical timbre and capture the physical attributes that are perceptually relevant for describing each musical instrument. Research based on perceptual judgments of natural or manipulated notes as well as space modeling using multidimensional scaling (MDS) [15] support a contribution of both spectral and temporal cues [3, 7, 16]. In a recent study, we have in fact corroborated such observation and argued that the spectrotemporal coding of sensory features in the mammalian auditory system, particularly at the level of auditory cortex, provides a neural basis for the representation of both spectral and temporal acoustic attributes relevant for timbre perception. A neuro-computational model based on spectro-temporal receptive fields, mimicking cortical tuning properties, is able to correctly identify each instrument from a database of isolated notes of 13 instruments over a wide range of pitches with an accuracy as high as 98.7 % [17].

That being said, the physical characteristics of a musical instrument are greatly shaped by the context of a musical phrase. Just like phonemes in speech are shaped by coarticulation, prosody and phonological structure of the syllable, word or utterance in order to convey a linguistic message; musical notes are also markedly affected by the melodic language of a musical piece. The acoustic manifestation of musical notes is greatly shaped by the melodic line, rhythmic structure, tempo as well as playing style and musical genre. This effect is more prominent in the temporal properties of notes, which affect the presence, absence, and duration of the attack, sustained portion of the note, dynamics within each note as well as transition between notes [14, 18, 19]. Modulation of the dynamic nature of notes is accompanied by changes to the spectral profile of the note causing variability in the expected shape and details of the spectrum relative to an isolated note. The recording or playing environment will also affect the acoustic characteristics of the waveform; though that is not exclusive to a musical piece and can also manifest itself with isolated notes.

This variability clearly complicates the problem of automated musical instrument identification. Generally, machine systems aim to extract informative features from the acoustic signal to obtain a good description of the multidimensional space of musical timbre. Attributes based on spectral envelope, temporal envelope, Mel-Frequency Cepstral Coefficients (MFCC), Linear Predictive Coding (LPC), statistical moments along time and frequency are commonly used for tasks of instrument identification, categorization, and indexing [20–23]. These features are typically combined into a vectorized representation of the timbre space that is either analyzed as a function of time or contains summary statistics for a short-time window or a given musical note. The applicability of this vectorized representation for isolated notes and musical phrases varies widely across studies [24–29], particularly because the acoustic manifestation of each instrument from isolated note to full melodies may or may not be well captured by the chosen spectrotemporal features extracted from the signal itself.

The current work explores the relevance of the intricate spectrotemporal receptive field (STRF) feature space believed to capture neural underpinnings of musical timbre representation [17] for instrument identification in solo performances. The original model was developed and tested using a rich database of isolated notes with an average of 1980 notes per instrument (RWC database [30]). The advantage of exploring the physical space of musical notes using a database like RWC is that it provides a rich, diverse, and comprehensive scan of musical instruments playing their full range of pitches, with different playing styles and various physical instruments under a controlled recording environment one pitch at a time. One can then capitalize on this wealth of organized musical information to provide a complete mapping of the spectral, temporal, and joint spectrotemporal characteristics of each instrument. The timbre space learned from this database needs to then be carefully tapped into in order to explore the overlap between the space based on isolated notes and a corresponding space capturing notes in the context of a solo performance. Here, we explore the short-term analysis of solo pieces and their correspondence to the musical timbre space, as well as a careful sampling of the musical phrase to best track the evolution of the musical phrase across notes and benefit from the learned knowledge based on isolated notes. We also investigate adaptive clustering techniques to map from one space to the other. In choosing solo musical performances, we focus on musical content from real world solo recordings obtained from commercial Compact Discs (CD) with no a priori screening.

Section 2 provides details about materials and methods used to setup our recognition system. These include the datasets used (subsection 2.1), approaches to parsing continuous solo recordings (subsection 2.2), methodology for analyzing acoustic signals using STRF features (subsection 2.3) as well as the setup of training and testing our classifier (2.4). The evaluation section 3 details the outcome of instrument-recognition experiments using isolated notes, musical phrases in a mixed training/testing setup using both STRF features and comparative approaches. A number of follow-up analyses follow in subsection 3.4 to investigate tests using artificial datasets and various feature sets that shed light on the nature of the discrepancy in musical timbre characteristics between isolated notes and continuous phrases. Subsection 3.5 directly estimates the degree of mismatch between these two datasets using information theoretic measures. Finally, subsection 3.6 proposes a potential resolution to the issue of mismatch by using adaptive classification techniques that circumvent the divergence in statistical characteristics between the two datasets. A discussion (section 4) summarizes the main findings of the study and remarks on the empirical findings regarding cross training from isolated to continuous notes.

## 2 Materials and methods

### 2.1 Datasets

Instrument recognition of solo recordings is tested in two main databases: the RWC database [30] which consists of isolated music notes with varying playing styles; and a collection of solo performances from commercial compact discs (CD), consisting of about 2 h of data per instrument (see Appendix for details on pieces included in the current study). The choice of CDs used in this study is completely arbitrary, solely based on availability, and is not pre-screened in any way. In the current study, we focus our analysis on six instruments: piano, violin, cello, saxophone, flute, and clarinet; for which we could collect a reasonable amount of solo performances. All sounds are downsampled to 16 kHz and pre-emphasized with a FIR filter with coefficients [1, −0.97].

### 2.2 Parsing solo recordings

In dealing with continuous solo recordings, the musical phrase needs to be properly segmented. Here, we explore two possible techniques: (1) a uniform windowing technique where each audio is segmented into non-overlapping regions of duration *τ_w_*; (2) a note extraction procedure that identifies the possible transitions between notes in the musical phrase. Both approaches are detailed next.

#### 2.2.1 Windowing segmentation

The windowing approach is the least computationally costly technique to process a continuous recording. It involves segmenting the signal into non-overlapping windows of duration *τ_w_*, which are subsequently analyzed through the cortical model to yield a spectrotemporal representation of the signal, averaged over its duration *τ_w_*. This method ignores the occurrences of notes or chords and treats each segment equally. The window duration, *τ_w_*, is a parameter that controls the time span of the features extracted from the signal, and can therefore play a crucial role in matching the features from solo performances relative to features extracted from isolated notes. The final choice of window duration *τ_w_* used in the current work is found empirically by choosing the duration that yields that best recognition performance across our solo and RWC datasets (see section 3).

#### 2.2.2 Harmonicity-based segmentation

The alternative way to a uniform sampling of the solo performances is to extract the individual notes in the phrase itself. Traditionally, the task of note extraction is often morphed into a task of onset detection, where a note is defined as the region between two onsets. Onsets are caused by a break in the steady state nature of a note, and onset detection involves evaluating a given audio signal using an onset detection function and applying certain selection criteria to decide the onset times. Phase deviation features have been widely used to detect departure from steady state behavior of a note, and hence, successfully applied to onset detection [31, 32]. However, onset-based techniques are quite sensitive to signal level characteristics which are easily affected by changing conditions like recording instruments and environments. They also require tedious tuning of parameters and thresholds for detecting transitions that vary greatly across databases [31]. Applying this approach in the current work is indeed challenging given the uncontrolled nature of the commercial CD recordings used here requiring different tunings of thresholding criteria on a per recording basis.

Instead, we opt for a harmonicity-based parsing method. Each note is typically characterized by a region of relatively steady pitch and significant harmonicity level. Here, we use this steady-state information to identify regions of stable pitch frequency and high harmonicity. The analysis starts by a pitch estimation using a template matching approach as proposed by Goldstein et al. [33]. The spectrum (or spectral slice of the spectrogram) at any given time frame is compared to an array of pitch templates. These templates represent the auditory spectrum of a generic note at a particular fundamental frequency. Here, we generate pitch templates *T*(*f* ; *f_p_*) as a cosine function modulated by a Gaussian envelope repeated at the integer multiples of a fundamental frequency *f_p_* as given by Eq. 1.

(1) $$T(f;f_{p})=\sum_{n}2e^{-{\left(\frac{f-{\text{nf}}^{p}}{\alpha \theta (n)}\right)}^{2}}\text{ cos}(2\pi \frac{\theta (n)}{\beta }(f-{\text{nf}}_{p}))$$

where *θ* (*n*) = 1 + 0.7 * *n* is a shrinkage factor, *α* = 18.28 and *β* = 26 are constants. We use 128 pitch templates spanning 5.3 octaves; which gives a resolution of one template every half semitone. The spectral slice of the spectrogram at every given time *y*(*t*_0_, *f*) is compared against the template at each pitch frequency *f_p_* generating a range of correlation values *ρ*(*t*_0_; *f_p_*). The template with the maximum match is chosen as the corresponding pitch value for the spectrum at time *t*_0_ and the degree of match captured by the harmonicity variable *H*(*t*) (see Eq. 2).

$$\rho (t;f_{p})=\text{corr}(y(t,f),T(f;f_{p}))$$

(2) $$P(t)=\text{arg }\underset{f_{p}}{\text{max}}\;\rho (t;f_{p})$$

$$H(t)=\underset{f_{p}}{\text{max}}\;\rho (t;f_{p})$$

where *y*(*t*, *f*) is the spectrogram derived in Eq. 5 and *corr* is Pearson’s correlation coefficient. The harmonicity *H*(*t*) indicates the degree of match to the template at the selected pitch value *P*(*t*). Based on this metric, we define a transition between notes as the region with change of pitch over time, accompanied with a reduced harmonicity value (due to possible overlap between notes at the boundary or percussive components in the onsets of the notes such as the hammer in the piano or bowin the violin). We define note boundaries using both pitch and harmonicity functions by setting selection criteria.

Note boundaries are selected based on the pitch function *P*(*t*) when the following condition is met:

(3) $$|P(t)-\text{mode}\{P(t-w),P(t-w+1)\ldots P(t)\}|\geq \tau_{1}$$

where *τ*_1_ = 0.2 and *w* = 30 *ms*. Note boundaries can also be selected based on the harmonicity function *H*(*t*) (which is normalized to be 0 mean and unit variance) when the following three criteria are satisfied:

H(t)≤H(k),∀k:t−w≤k≤t+w

(4) $$H(t)\leq \frac{\sum_{k=n-\text{mw}}^{n+w}H(k)}{\text{mw}+w+1}-\tau_{2}$$

$$H(t)\leq g_{\mu }(t-1)$$

where *m* = *w* = 30*ms*,*τ*_2_ = 0.3, *mu* = 0.1, and *g_μ_* (*t*) = min(*H*(*t*), *μg_μ_*(*t* − 1) + (1 − *μ*)*H*(*t*)).

**Fig. 1:**
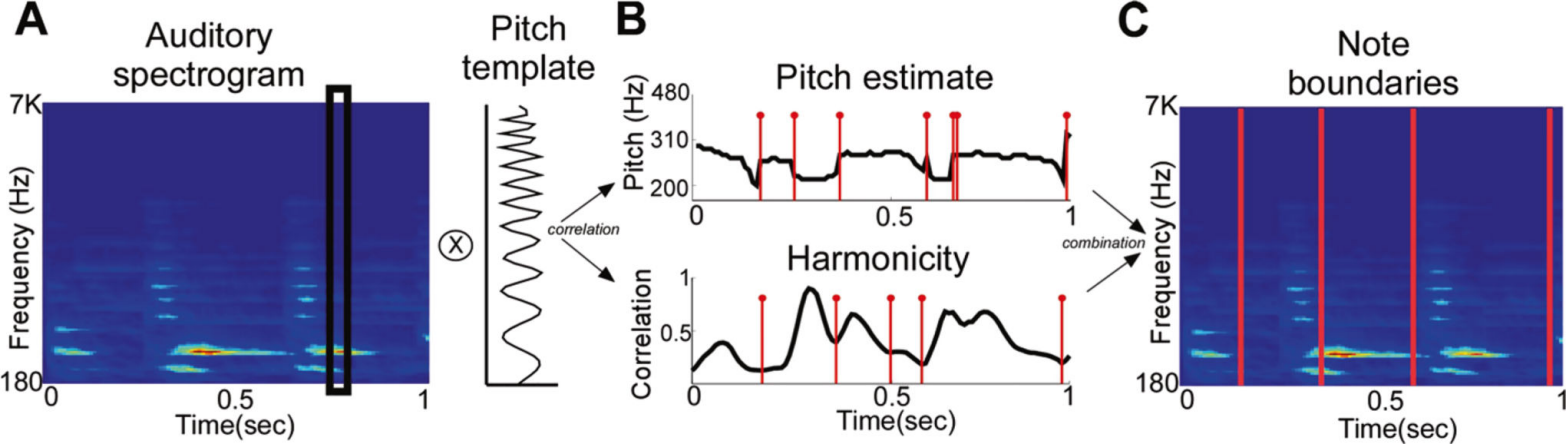
Note extraction scheme. An example of a spectrogram (**a**) of a piano audio segment containing four notes which is convolved with a pitch template to yield the (**b**) pitch estimate and harmonicity along with the candidate onset points. Finally, the note boundaries (**c**) are depicted in red where the candidates coincide

Finally, the actual segmentation boundaries are selected as those times where the potential boundaries based on both the pitch *P*(*t*) and harmonicity *H*(*t*) agree (with a tolerance of 40 ms) (Fig. 1).

The note segmentation method described above is not error proof. One of the main sources of erroneous parsing of the solo recordings in the presence of simultaneous notes (i.e., chords). Chords cause the harmonicity estimate to yield a large number of shorter segments with relatively stable pitches. To deal with this potential source of error, we confine our analysis to notes extracted that are longer in duration than a minimum threshold *τ_n_*, defined empirically based on classification accuracy (see section 3). We also contrast the harmonicity-based segmentation described here to an onset-based method commonly used in the literature. Here, we test the “Rectified Complex Domain” (RCD) approach proposed by Dixon [31]. This onset-detection method is implemented as described in the publication with a 2048 hamming window and shift of 441 sample (corresponding to 46 ms at a sampling rate of 44100 Hz). The Short Term Fourier Transform uses a shift of 10 ms. The onsets from the RCD function are calculated using the parameters suggested in the paper (*ω* = 3, *m* = 3, *δ* = 0.5 and *α* = 0, see [31]).

It is important to note that the harmonicity-based method described here is not a complete note segmentation approach in its own right. The technique simply relies on the steady-state behavior of pitch information that is typical in each musical note, and detects changes in this steady-state character in order to delimit potential transitions to a new note. It does not carefully track onsets and offsets of each note nor is it able to properly parse irregular patterns such as instruments with long attack times (e.g., flute). It is likely that harmonicity does complement a number of signal-based techniques (using envelope or phase information) to provide a more robust acoustic-based partitioning of a solo musical phrase.

### 2.3 The STRF feature space

**Fig. 2:**
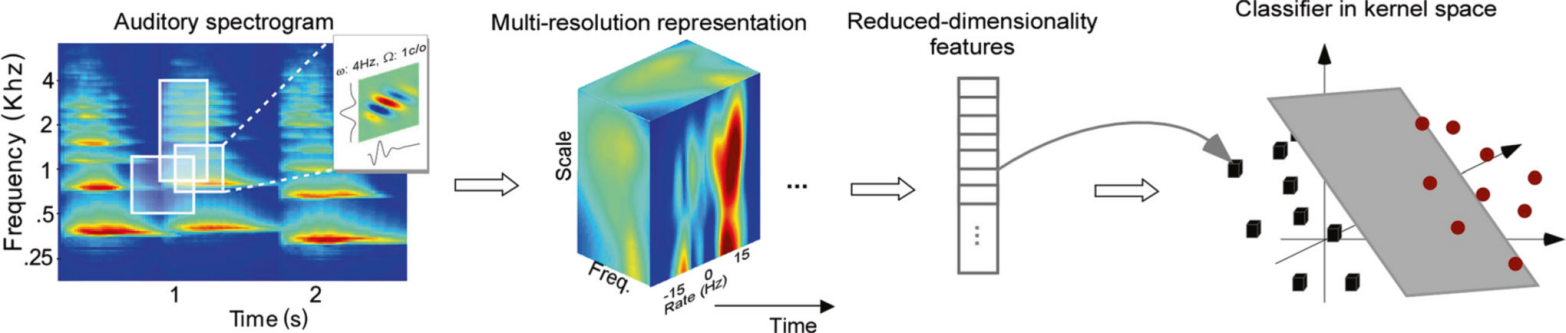
Schematic of the STRF-based instrument classification. Schematic of the processing stages involved in the STRF-based model of instrument classification. A time-frequency spectrogram is derived for each acoustic signal, then further mapped onto a higher dimensional space using an STRF-based model. The STRF space is then reduced in dimensionality and mapped via a kernel function to a new space to define boundaries between different musical instruments

All signals are analyzed using a model developed to explore the neural underpinnings of musical timbre [17]. The model performs a decomposition of the spectrotemporal modulations of the acoustic signal. Modulations reflect the “changes" or variations in the spectral profile (e.g., peaks, troughs, center of gravity, smoothness of the spectrum) as well as “changes” or variations in the temporal structure (e.g., rise and fall of the temporal envelope, onsets, periodicity patterns). This level of detail results in a intricate analysis of the signal characteristics in a multi-resolution mapping, believed to mimic the filtering properties reflected by neurons in primary auditory cortex. Here, we review the key transformations in the model and point readers to [17, 34] for further details. Figure 2 depicts a schematic of the key stages in the model.

The initial stage of the model maps the one-dimensional acoustic waveform *x*(*t*) onto a two-dimensional time-frequency representation *y*(*t*, *f*). This transformation starts by convolving *x*(*t*) with a bank of 128, highly asymmetric, constant-Q filters *h*(*t*, *f*) organized on a logarithmic axis spanning 5.3 octaves. This stage models spectral filtering at the level of the cochlea and is followed by additional spectral sharpening modeled as a derivative along the frequency axis, and subsequently by a half wave rectification. Finally, the loss of phase locking at the midbrain level is modeled as a low pass filter $L(t,\tau )=e^{-(\frac{t}{\tau })}u(t)$, where *u*(*t*) is the step function and *τ* = 4 *ms* is a time constant. These transformations yield a two-dimensional auditory spectrogram that is further enhanced using a cubic root compression to boost low amplitude events and transitions (Eq. 5). This spectrographic representation of sound tracks the spectral profile of the signal as well as the temporal “envelope modulations” due to interactions between spectral components that fall within the bandwidth of each filter. The frequencies of these modulations are naturally limited by the maximum bandwidth of the cochlear filters. The resultant auditory spectrogram can be easily replaced by other time-frequency representations (e.g., Short-Term Fourier Transform, Slaney’s Gammatone toolbox spectrogram [35], etc). The biologically-inspired representation chosen in the current study (Eq. 5) has been shown to exhibit interesting properties such as self-normalization and robustness [36].

(5) $$y(t,f)={\left[\text{max}(\partial_{f}x(t){⊗}_{t}h(t,f),0){⊗}_{t}L(t,\tau )\right]}^{\frac{1}{3}}$$

The next stage further decomposes components of the spectrogram through a bank of modulation-tuned filters *𝒢*, selective to specific ranges of modulation in time (rates *r* in Hz) and in frequency (scales *s* in cycles/octave), called STRFs (spectro-temporal receptive fields). The STRF filters are defined by:

(6) $${𝒢}_{+}(t,f;𝔯,𝔰)={𝒜}^{*}(h_{r}(t;𝔯))𝒜(h_{s}(f;𝔰))$$

$${𝒢}_{-}(t,f;𝔯,𝔰)=𝒜(h_{r}(t;𝔯))𝒜(h_{s}(f;𝔰))$$

where 𝒜(․) indicates an analytic function, (․)* is complex conjugate, and +/− indicates upward or downward orientation selectivity in time-frequency space (i.e., detecting upward or downward frequencies sweeping over time). The use of the analytic and complex conjugate pairing ensures that the receptive field are complex functions that share quadrant-separability properties observed in physiological data. In other words, these wavelet functions are not a simple separable product of a spectral and a temporal function (see [34] for further discussion). The seed functions *h_r_*(*t*) and *h_s_*(*f*) are shaped as Gamma and Gabor functions respectively, as given in Eq. 7.

(7) $$h_{𝔯}(t)=t^{3}e^{-4t}\text{ cos}(2\pi t),h_{𝔰}(f)=f^{2}e^{1-f^{2}}$$

and their scaled versions are given by *h_r_*(*t*; 𝔯) = 𝔯*h_r_*(𝔯*t*) and *h_s_*(*f* ; 𝔰) = 𝔰*h_s_*(𝔰*f*).

The final output of this STRF-based analysis is then a four-dimensional complex-valued representation along time *t*, frequency *f*, temporal modulations 𝔯 and spectral modulations 𝔰; given by:

(8) $$𝒵(t,f;𝔯,𝔰)=y(t,f){⊗}_{t,f}𝒢(t,f;𝔯,𝔰)$$

In the current study, we use 11 temporal rates equally spaced on a logarithmic axis from 4 to 125 Hz in both upward and downward directions, and 11 spectral scales equally spaced on a logarithmic axis from 0.25 to 8 cycles/octave. We also average the magnitude of the modulation representation 𝒵 along time over the duration of the signal (i.e., entire musical note in case of RWC databaset) or analysis window (see discussion of choice of time window below), and further reduce the dimensionality of the 22×11×128 STRF tensor to a 420 dimensional vector **X**_*i*_ using tensor singular value decomposition [37] preserving 99.9 % of the variance along each dimension. It is important to note that instead of analyzing the signal over short-time windows and maintaining a time series representation over all windows, the current approach averages across the entire duration of the signal being analyzed and maintains only average statistics. While time is not explicitly represented, it is implicitly captured via the temporal modulation axis (𝔯) which captures how the signal changes over time, hence effectively encoding information about the temporal envelope of each spectral component in the acoustic waveform.

### 2.4 Recognition setup

Finally, the reduced-dimensionality feature vector **X**_*i*_ ∈ ℝ^420^ is combined with its instrument label **Y**_*i*_ ∈ (+1, −1) to forma training dataset ${𝒟}^{420}={\left\{(X_{i},Y_{i})\right\}}_{i=1}^{N}$ for a classifier that distinguishes pairs of instruments labeled as {+1} or {−1}, where *N* is the total number of available data vectors. Here, we use a standard support vector machine classifier with radial basis functions [38]. Effectively, the classifier learns a mapping, or decision function:

(9) $$𝒥(X):\;{\mathbb{R}}^{420}\to \{+1,-1\}$$

(9) $$𝒥(X_{i})=w^{T}\phi (X_{i})$$

Here, *ϕ*(․) is the radial basis kernel chosen for this study, and w is a linear decision boundary derived by optimizing the following function:

(10) $$\underset{w}{\text{min}}\frac{1}{2}{\left\|w\right\|}^{2}+C\sum_{i=1}^{N}\xi i$$

such that *ξ_i_* ≥ 0, **Y**_*i*_w*^T^ ϕ*(**X**_*i*_) ≥ 1 − *ξ_i_*, ∀(**X**_*i*_, **Y**_*i*_) ∈ 𝒟^420^

*C* is a scalar cost factor and $\sum_{i=1}^{N}\xi i$ measures the total classification error. Essentially, the classifier identifies boundaries between classes of instruments. We train pairwise classifiers for every pair of instruments, and use the winner as the selected class across all pairwise comparisons. In the current work, the training and testing data are extracted from one of three possible sets: (1) matched setting: both training and testing data are from the same database (either RWC or solo recordings parsed in a specific manner); (2) cross-domain setting relative to RWC: the training data for the classifier is defined from RWC notes while the testing data is extracted from a parsing of the solo recordings; (3) cross-domain setting relative to solo: the training data is compiled from a parsing of the solo recordings while the testing is performed on the RWC notes. In the matched setting, we use different data subsets for training and testing. In all cases, we perform a grid search to tune the optimal choice of classifier and kernel parameters, and use a ten-fold cross-validation to evaluate the performance of the system. Accuracy is defined as the sum of correctly classified examples from all instruments divided by total number of examples from all instruments. Examples refer to solo notes, windows or isolated notes, depending on the specific experiment. All instruments were given equal weight in this computation.

## 3 Evaluation

### 3.1 Uniform windowing of solo recordings

**Fig. 3:**
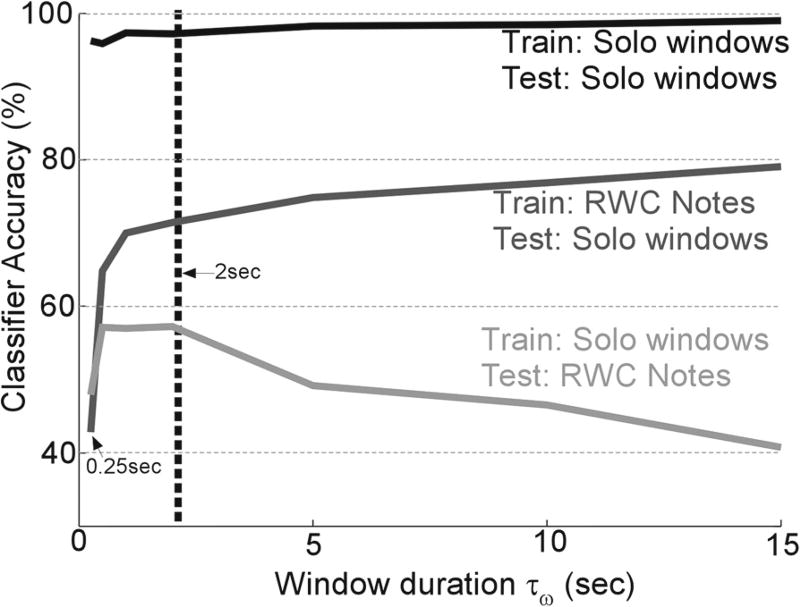
Recognition accuracy using uniform windowing of solo performances. Accuracy for uniform windowing experiments in matched and cross-domain train/test settings of continuous solo phrases as a function of window size *τ_w_*

In order to determine the optimal choice of uniform window length *τ_w_* for parsing the solo recordings, we performed three sets of recognition experiments based on a matched setting (train on solo, test on solo) and cross-domain setting (train on solo, test on RWC or train on RWC, test on solo). The RWC notes are analyzed one note at a time (averaged over the entire duration of the note), while the solo recordings are parsed into segments of length *τ_w_* then analyzed through the receptive field model. Figure 3 shows the tradeoff between short and long-term spans of the analysis window *τ_w_*, as a function of accuracy of our recognition model. The best performance is achieved in a matched context where training and testing is done on a uniform set of segmented solo windows. In this case, the classifier quickly saturates as *τ_w_* grows from as low as 250 msec to few seconds and seems to depend very little of the value of *τ_w_*. In contrast, training and testing with a mismatched dataset is greatly affected by the window duration. Training on RWC notes and testing on solo segments quickly improves for short segments and hovers between 70–80 % accuracy with a monotonic increase. In the opposite setting, training on solo segments with very short windows (e.g., 250 msec) or too long windows seems to greatly affect the performance. Shorter segments likely capture too much variability in the instrument’s time profile hence producing inconsistencies in the features learnt from each class, for instance confounding the transient and steady-state nature of the signal. Longer windows excessively average the temporal profile of each instrument making it harder to distinguish from instruments with comparable spectral profiles. A balance between short-term and long-term averaging appears to peak around 2 s. In the current study, we choose *τ_w_* = 2 *s* as our optimal choice for all future experiments. Clearly, this choice can be optimized for different applications, and is likely affected by the diversity in the solo database used. It may also be slightly biased by the comparison with the RWC database, whose notes are on average 2.7 s in duration, though they varied between 0.1–18 s.

### 3.2 Harmonicity parsing of solo recordings

**Fig. 4:**
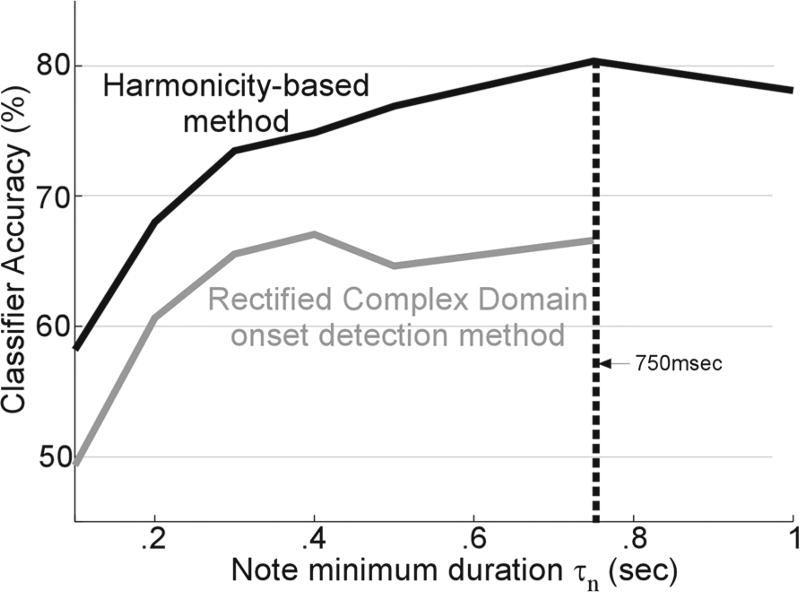
Harmonicity-based note extraction accuracy. The plot depicts the accuracy of the classifier on isolated notes extracted using a harmonicity-method, as a function of minimum note duration *τ_n_*. This method is contrasted with an onset-based method (the rectified complex domain, by Dixon [31]) for note extraction from musical phrases

In order to test the use of harmonicity-based parsing of solo recordings, we ran a recognition experiment in a cross-domain setting (train on RWC, test on solo). We empirically test for the optimal choice of minimum note duration as derived by the harmonicity parsing. Only notes extracted with duration at least *τ_n_* are analyzed. Figure 4 shows the classifier-accuracy improvement as a function of minimum note duration. This accuracy peaks around 750 msec before it starts dropping again. The optimal choice of 750 msec is not necessarily reflective of a fundamental tempo or window size in the data. Rather, it is constrained by the total amount of data we have available for our solo recordings database. Constraining the note to be of a certain duration limits the number of notes we have available in the database. Based on the performance of the note extraction shown in Fig. 4, we choose 750 ms as the value of *τ_n_* for all future experiments. Overall, the harmonicity-parsing algorithm suggests that our selection of solo CDs include an average of 7569 notes per instrument with mean duration of 0.44 s and median of 0.26 s per note (ranging from 0.1 to 4.85 s).

We also contrast the harmonicity-based method with other note extraction techniques from the literature based on onset-detection. Figure 4 overlays the performance of the same support vector classifier optimized for notes extracted based on onset-detection following the Rectified Complex Domain approach proposed by Dixon [31]. As is evident from the classification results, the pitch-harmonicity measure allows for more accurate identification of musical instruments for all values of *τ_n_*, irrespective of pruning based on acceptable note size.

### 3.3 Instrument recognition results

To fully explore the relevance of the modulation feature space in capturing informative characteristics of musical timbre, we use the model with the chosen solo parsing parameters (for uniform windowing and harmonicity-parsing) to test classification accuracy in a matched and cross-domain setting. Table 1 shows a ten-fold cross-validation contrasting three classifiers, each trained on one of the three sets (RWC, harmonicity-parsing notes and uniform windows). All three sets yield a high performance above 97 % in a matched training-testing. The performance drops when a mismatched set is used for training and testing.

Taking a close look at the results from Table 1, we note that the mixed training/testing on solo recordings using uniform windows or segmented notes reveal a high degree of agreement across both methods. The higher accuracy for the harmonicity-parsing technique as compared to the uniform windowing technique when tested against a classifier trained on RWC notes indicates that note extraction based on harmonicity was better at reducing the difference between the datasets. This result is not surprising since the RWC dataset also has isolated notes. Finally, the low classification accuracy for the classifiers trained on the feature sets derived from solo music database when tested on RWC database indicates that RWC database is a more generalized database with much more variance in the data as compared to the solo music database collected for the current study. This outcome could potentially be improved with inclusion of a larger dataset of solo recordings.

In order to provide a comparative reference of the performance of the STRF feature space relative to other existing approaches, we rerun the same set mixed training/testing classifications using audio features from the MPEG-7 audio framework which include zero-crossing, spectral slope, spectral roll-off as well as spectral envelope features resulting in 260-dimensional feature mapping. No temporal moments are included in the analysis. These features are extracted for each analysis segment (entire note duration in case of RWC notes, fixed window size in case of uniform sampling of solos, parsed notes in case of harmonicity parsing of solos) then averaged over the entire duration of the segment in a similar fashion as the time-averaged STRF features. Such MPEG7 features were recently used as front-end for a number of automatic classification tasks for audio and musical instruments combined with various classifiers, including non-negative matrix factorization [39, 40]. Here, we test these MPEG7-based features with our support vector machine classifier using a similar mixed training/testing setup as used for the STRF features. Table 2 shows a drop in performance across all testing conditions when using these MPEG7-based features. While this drop in performance is not a definitive statement of the superiority of the STRF approach, it reflects that these two methods capture different levels of granularity in the signal, which provide different sets of informative features to a back-end classifier.

In a separate experiment, we investigate how the classification system with solo recordings behaves with “unseen” data. We extract note segments from all but one CD (selected at random for each instrument) using the harmonicity-based parsing approach. This data is then divided into a 90 % training set and 10 % testing set (homogeneous test). The same classifier trained on 90 % of the data is tested again with data from the left out CD (heterogeneous test). Table 3 summarizes the classification results using both STRF and MPEG7 features. Using both feature sets, the performance does drop, though in a more dramatic fashion in the case of MPEG7 features. Note that the CD selection was not pre-screened in any way, and the selection included a wide range of recording settings and playing styles that are difficult to capture when training on a small number of CDs (as little as 1 CD in case of piano for example). Nevertheless, the accuracy remains at a high level that could certainly be strengthened with enough diversity in the training set.

Finally, we perform an additional experiment to explore the contribution of different acoustic features. Our earlier study [17] explored the contribution of both spectral and temporal dimensions; and has indeed confirmed that the use of joint spectro-temporal modulation features is key to fully accounting for the multidimensional nature of musical timbre in isolated notes, in agreement with earlier findings in the literature [20]. To complement these previous observations, we compute the performance of our classifier on RWC notes as well as isolated solo notes using the harmonicity-based parsing approach with varying combinations of acoustic features (frequency, scale, and rate). Table 4 shows the classifier accuracy for different feature combinations. The results confirm a number of observations: (1) frequency is an important dimension in defining instrumental timbre; (2) augmenting the frequency axis with rate or scale dimensions provides improvement to the classifier accuracy; (3) including all three dimensions of rate, scale, and frequency further improves the accuracy results on solo notes.

### 3.4 Follow-up analyses

We run follow-up tests to better understand the correspondence between isolated notes and notes in continuous solo performances. These follow-up analyses use artificial datasets recreated from the datasets used in the main study. First, we create a new dataset by concatenating notes along time from RWC database in order to simulate the succession of notes in a solo musical phrase. To determine the number of notes to be concatenated, we compute a histogram of the number of notes that are extracted from 2-s segments. The histogram yields the values [58, 32, 8.5, 1, and 0.5 %] where the first number indicates the ratio of single notes, the second indicates the number of times two notes were extracted and so on. An artificial dataset with 2000 samples per instrument class is then created by concatenating the required number of notes, randomly selected, to match this histogram. We then train a classifier on this artificial set and test on uniform windows from solo music dataset. This experiment yields an accuracy of 71.76 %. The lack of significant improvement, when compared to the model trained on RWC notes (71.42 %), suggests that artificial concatenation of isolated notes does not recreate the transition characteristics between notes in a musical performance, and hence provides no further improvement in matching uniform solo segments with isolated notes from RWC.

Second, we consider the type of mismatch that occurs due to the presence of chords in the solos. Specifically, we are interested in probing whether our parsing of notes from solo music mistakenly misses instances with musical chords that are labeled as clear notes. We artificially simulate chords in the training set, by overlapping two randomly selected notes in time to generate additional data for training. We use 1000 original notes from the RWC dataset and 1000 artificial chords per instrument to yield a new enriched training RWC dataset. The testing is performed on original windows from the solo dataset. A new model is then trained with this expanded RWC dataset and tested against uniform windowed segments resulting in a performance of 70.81 % accuracy. This chord-enriched training dataset does not significantly change the performance of the classifier with original RWC training/solo notes testing. Our results indicate no improvement in classifier performance. This suggests that the existence of few chords in the parsing of solo phrases is likely a negligible factor in explaining the mismatch between the two datasets. Indeed, the solo CDs used in our current analysis contain very few instances of chords. An informal listening test indicates that less than 5 % of the notes are chords. A more careful analysis using annotated musical performances will be needed to formally assess the effect of chords on instrument recognition in isolated vs. solo phrases. In the datasets used in the current study, chords appear to be an insignificant factor in explaining the mismatch between isolated and continuous notes.

**Fig. 5:**
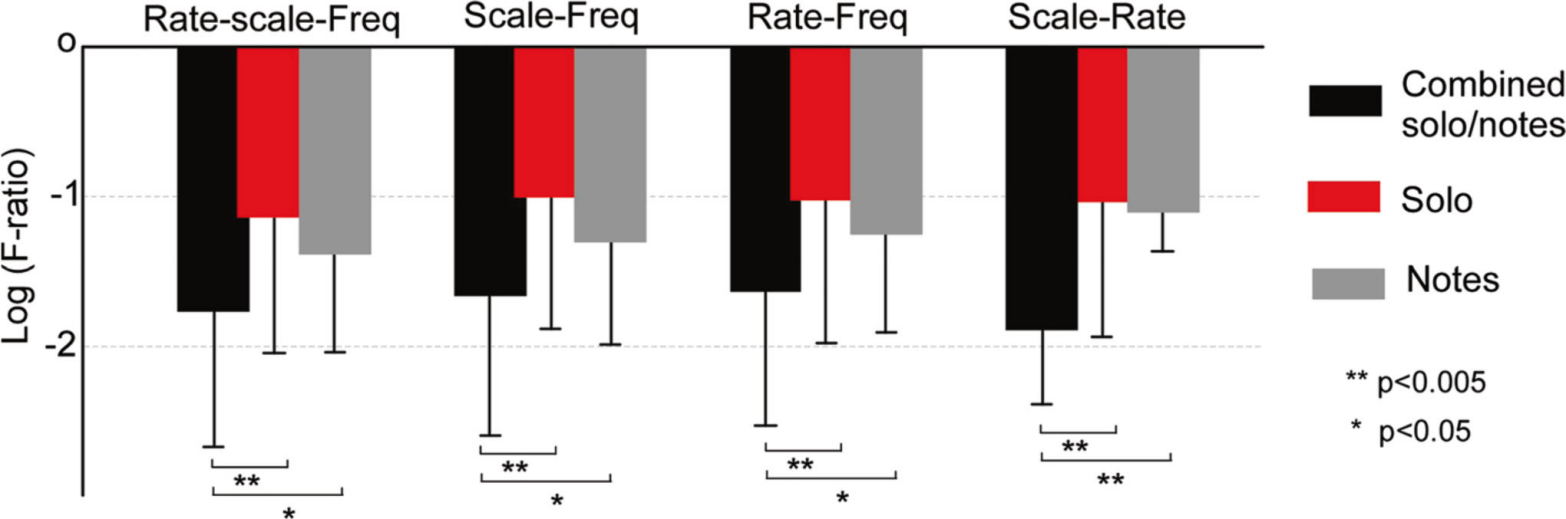
Fisher discriminant on combined solo and isolated notes for different feature sets. Average log F-ratio is depicted for each feature set; using rate-scale-frequency, scale-frequency, rate-frequency, and scale-rate. The analysis is performed on combined solo and isolated notes and tests the separability of this combined set to identify each instrument class. The *bar plot* shows the mean log F-ratio, *error bars* indicate standard deviation

Finally, to further investigate the mismatch between isolated notes and musical phrases in the temporal domain, we leave out temporal information in the STRF feature space by averaging the temporal modulation axis 𝔯) and only maintaining the scale-frequency dimensions *z*(*f*; 𝔯, 𝔰). These spectral-only features are then used to test a recognition system trained on notes extracted from solo recordings (using the harmonicity approach) and tested on isolated notes. This experiment yields an accuracy of 43.3 % (compared to 44.7 % when trained with full STRF features—see Table 2). Discarding temporal information does not seem to have a notable impact on the classification accuracy. This minimal change in accuracy score suggests that the mismatch (or lack thereof) of temporal characteristics between solos and isolated notes does not explain the accuracy of 44.7 % when testing on isolated notes. This low accuracy may be due to other factors (e.g., inaccuracy in parsing notes from solo signals, differences in transient or steady-state behavior of notes in a phrase which alters their spectral characteristics, or complete mismatch in temporal characteristics which causes no difference whether a temporal axis is included in the feature set or not). We confirm this observation by analyzing the homogeneity of different instrument classes using different acoustic attributes. To do so, we use the F-ratio (an extension of Fisher’s discriminant [41]) to assess discriminability across instrument classes [42]. Fisher’s discriminant classically operates on a two-class problem and measures the difference between the means or centroids of two classes relative to their variances. The F-ratio extends this definition to a multiclass problem. It is defined as the variance of means (between class) / mean of variances (within class). We combine a dataset using (randomly chosen equal portions of) solo windows and isolated notes and compute the F-ratio using rate-scale-frequency vs. scale-frequency features. The results highlight that combining the two datasets increases instrument mislabeling, hence significantly reducing class discriminability which is indicative of higher heterogeneity across the two datasets (Fig. 5). Moreover, in agreement with the classification outcome, the mean log F-ratio (we take the log value to highlight lower F-ratios) using rate-scale-frequency is −1.8 ± 0.7 while that using scale-frequency is −1.7 ± 0.7. Clearly, dropping the rate information does not significantly affect the separability across instruments when comparing a combined dataset of solo and isolated notes. In contrast, the same feature set (rate-scale-freq) appears to be more discriminative (higher average F-ratio) for more homogeneous datasets using solo or isolated notes by themselves (Fig. 5). Overall, an analysis of discriminability of rate, scale, and frequency features over individual datasets (solo, RWC) shows a higher separability of instrument classes within each dataset. In order to shed light on the heterogeneity of the feature space across solo and isolated notes, one has to take into account the sources of variability in the combined dataset, as analyzed next.

### 3.5 Discrepancy between datasets

In an attempt to directly estimate the differences between the isolated notes and solo recordings datasets, we compute the balanced Kullback-Leibler (KL) divergence [43] on distribution of features *z*(*f* ; 𝔯, 𝔰) for each instrument extracted from the two databases. The KL metric is a comparison of two probability distributions of a given instrument from both databases; defined as:

(11) $$\text{KL}(p_{1},p_{2})=\sum_{x}p_{1}(x)\text{ log}\frac{p_{1}(x)}{p_{2}(x)}+p_{2}(x)\text{ log}\frac{p_{2}(x)}{p_{1}(x)}$$

**Fig. 6:**
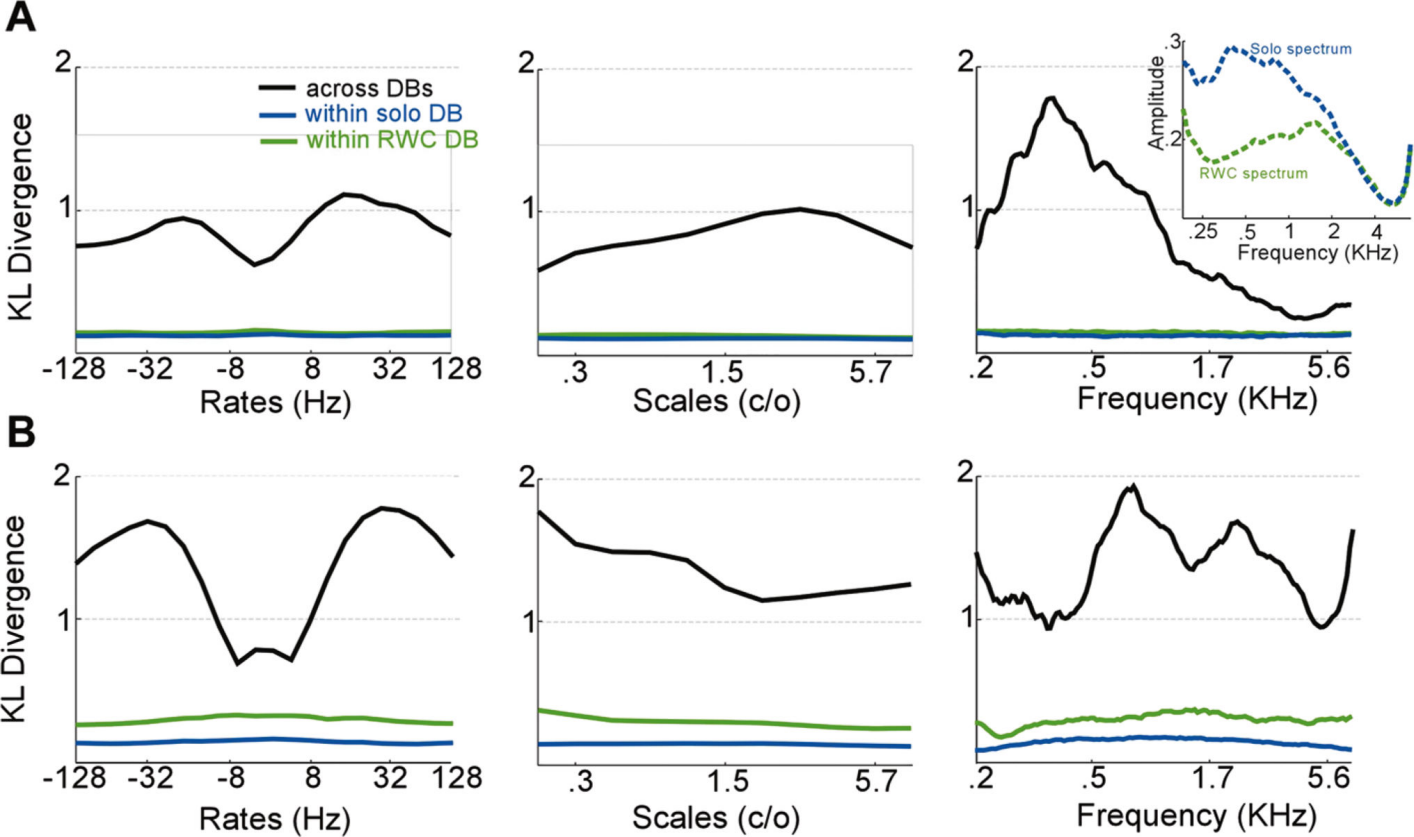
Average KL Divergence for piano and flute notes. **a** The average KL divergence between RWC notes and solo notes for piano is computed along the temporal modulation or rate dimension (*left*), spectral modulation or scales (*middle*), and frequency (*right*). *Inset* in right panel is average spectrum of RWC notes and solo notes. **b** Similar distance metrics for flute notes

This comparison gives us a better insight into the the main areas of mismatch between isolated notes in RWC dataset and notes extracted from the continuous recordings. We analyze this distance metric for each point along the three-dimensional space of rate-scale-frequency. Figure 6 shows the KL divergence averaged along each of the three dimensions for piano notes (Fig. 6a) and flute notes (Fig. 6b). For completeness, we compute KL divergence within pairs of signals from each database (RWC or solo performances) as well as comparing the two databases. As expected, the within database KL values are much lower and consistent across datasets suggesting a higher degree of consistency within the data from each set. In contrast, the RWC and solo notes show high degrees of disagreement at specific parts of the space depending on the instrument. For instance, the piano RWC and solo notes show greater discrepancy at lower frequency (< 1*KHz*). Examining the average spectrum from each database (inset in rightmost panel in Fig. 6a) confirms a different spectrum roll-off between the two datasets; which could be explained by a number of “music”-related factors such as resonance emphasis or “non-music”-related factors such as recording environment and channel distortions. Note that both datasets were pre-emphasized using a highpass filter with parameters [1, −0.97]). In contrast, the flute reveals a higher mismatch in the mid-high frequency range as shown in Fig. 6b, rightmost panel. Table 5 summarizes the regions of high divergence between the two datasets for all the instruments. This result highlights that discrepancies between the two datasets are not due to a systematic mismatch; but is rather instrument dependent. Teasing apart the causes of mismatch is not a straightforward endeavor. It could be due to many factors, including differences in recording instruments, room acoustics, channel noise, signal postprocessing and filtering emphasis, etc. A number of musical reasons could also contribute to this mismatch; notably due to the expressivity or transitions between notes in real recordings in contrast with isolated notes. Next, we explore a method to overcome the difference in distributions across instruments.

### 3.6 Adaptive cross-domain classifier

It is clear from the control and statistical analysis that the average profile distributions of segments from the solo and RWC databases play an important role in justifying the classification mismatch between the two datasets. In order to circumvent this divergence in signal properties, we investigate the use of an adaptation technique to adjust the support vector machine boundaries between instruments based on a first database to the new statistical profiles of a second different database using an adaptive SVM technique. When using RWC as our baseline training set, the current classifier learns a decision function 𝒥^*RWC*^(**X**) based on the profile of data **X** from the RWC notes (Eq. 9). In order to conform better to the statistical structure of the solo dataset, we use an improved cross-domain classifier, called an adaptive support vector machine. Essentially, a new decision function is learned, defined as: 𝒥^*solo*^(*x*) = 𝒥^*RWC*^(*x*) + Δ𝒥(*x*) where the new decision function follows a similar minimization procedure as a typical support vector machine classifier (Eq. 10) but with an added constrain to minimize the update term Δ𝒥 (*x*). This ensures that the decision boundary is kept as a close as possible to the original RWC-trained classifier. Details of the adaptation follow the exact procedure outlined in [44], using software provided by the authors of this work. By leveraging the knowledge from the available RWC database, this method makes small adjustment to the decision weight in feature space to accommodate the different distribution in the solo music. This procedure requires using small training data from the solo dataset. Without any adaptation, the support vector machine classifier trained on RWC and tested on solo recordings parsed using the harmonicity method yields an accuracy of 78 % (Table 2). We use 200 randomly selected notes from the solo music dataset per instrument as the adaptation set to adjust the decision boundary and retest the classifier with a separate set of solo segments (i.e., about 3–5 min of solo data). The performance of the model after adaptation is found to be 86.6 % indicating that we can successfully adapt a model trained on one dataset to another condition under limited data constraints. For this adaptation, we set the value of the cost parameter (C) to be 1 which was found to maximized the average performance across both solo music dataset and RWC notes.

## 4 Discussion and conclusions

The current work pursues the goal of musical instrument identification in continuous recordings. This problem combines the issue of both musical timbre recognition as well as dealing with the potential mismatch between readily available single music note data and continuous recordings. As is common in most systems of automated sound recognition, these issues translate to: (1) choosing appropriate signal characteristics and sound features that are most informative about the instrument class; (2) determining the relevant temporal context (e.g., choice of windowed analysis of the signal); and (3) adopting the proper statistical representation for correctly classifying the data.

In agreement with a number of findings in the literature using a variety of computational, psychophysical, and physiological explorations, it is clear that features that best capture the full complexity of musical timbre have to span the intricate space of time-frequency in a joint, synergistic way [3, 7, 20, 45–51]. The features explored in the current study attempt to provide a complete account of this complex spectrotemporal space, putting emphasis on the modulation patterns in the signal. This representation, inspired from neurophysiological recordings of single neurons in the primary auditory cortex, highlights not only the spectrogram-like features in the signal, but also how time and frequency trajectories change jointly along the temporal and spectral axes. This representation provides an indirect generalization of many features commonly used in the literature of timbre characterization [52], including envelope features, spectral shape and centroid, and temporal trajectories. One of the advantages of this representation as compared to more conventional features such as cepstral or predictive coefficients is its distributed nature along different time and spectral resolutions, capturing everything from broadband to narrow spectra, fast dynamics to slow temporal changes. Importantly, the space of spectral and temporal modulations is *jointly* represented which is a key attribute of any representation of musical timbre. While the approach using neurophysiological receptive fields does not come without its challenges (e.g., high-dimensional feature space, overly redundant representation), it is still able to perform remarkably in classifying musical instruments in a large database or selection of solo CDs, with accuracies 97 % and above.

While the applicability of such STRF features for continuous solo performances is remarkably accurate, the cross-domain transition from single notes to musical phrases (even for solos) is not a trivial one. As our analysis shows, the classic windowing approach to parse musical phrases is suboptimal [23, 26, 28, 53]. It coarsely bins the time signal into segments of equal length; but with no consideration to the underlying structure. A more effective way is to provide a better match to the composition of the musical phrase; by attempting a pitch-based parsing of individual notes. Ultimately, the recognition of musical instruments in continuous recordings is a delicate balance of acoustic, musical, and environmental factors. On the one hand, the physical attributes of each musical instrument color its sound with unique spectro-temporal features. These are best extracted by a rich enough feature set. On the other hand, the constrains of the musical genre, the melodic rhythm as well as the non-musical constrains (e.g., recording environment, choice of physical instrument, playing style, signal preprocessing) greatly shape the characteristics of the signal and ultimately the true match of the instrument’s identity. An analysis of KL-divergence between the RWC single notes database and the off-the–shelf solo CDs used in the current study highlights that differences are not feature specific (time vs. frequency) or instrument specific (Table 5). In addition, our follow-up analyses show that discrepancies in training/testing across datasets cannot be simply explained by issues with the parsing method such as note sequence structure or presence of chords. In order to best mitigate the divergence in statistical characteristics between the two datasets, the current work proposes the use of adaptive classification methods that maintain the structure of a model trained on isolated notes but regulate their decision boundary to capture diverging properties from continuous musical phrases. The use of this adaptive approach provides an improvement in recognition accuracy of about 6 % with very minimal training data using less than 5 min of additional data from the solo dataset.

Overall, while a true instrument identification system would have to carefully account for cross-referencing a variety of datasets [54], the current study sheds some light on the applicability of a rich STRF feature space and adaptive machine learning techniques. The exploration of different levels of abstraction could be greatly informative in addressing the mapping from single notes to continuous recordings. Considering the temporal placement of notes in the context of the entire musical phrase as well as para-timbral information could greatly inform the identification of the musical instrument; though it would take the system from a purely acoustic-driven analysis to a more data mining and information retrieval approach.

## Figures and Tables

**Table 1:** Results of cross-testing instrument recognition using STRF feature space

Train\test	RWC	Notes	Windows
RWC	98.5 ± 0.2 %	78 ± 2.1 %	71 ± 1 %
Notes	44.7 ± 0.9 %	97.7 ± 0.6 %	93.4 ± 0.5 %
Windows	58.5 ± 1.5 %	97.3 ± 0.5 %	96.9 ± 0.4 %

**Table 2:** Results of cross-testing instrument recognition using MPEG7-based spectral features

Train\test	RWC	Notes	Windows
RWC	62.2 ± 1.0 %	51.0 ± 1.4 %	43.1 ± 1.0 %
Notes	41.3 ± 1.3 %	79.3 ± 1.4 %	70.3 ± 0.7 %
Windows	38.6 ± 1.8 %	81.4 ± 1.3 %	78.4 ± 0.9 %

**Table 3:** Classification results using homogeneous training/testing or heterogeneous (leave one CD out) conditions

Features	Homogeneous test	Heterogeneous test
STRF features	97.7 ± 0.6 %	88.1 ± 0.5 %
MPEG7 features	80.0 ± 2.4 %	66.1 ± 0.6 %

**Table 4:** Classification results with matched training/testing using different acoustic features

	Rates (22dim)	Scales (11dim)	Freq (128dim)	RateScale (242dim)	ScaleFreq (420dim)	RateFreq (420dim)	RateScaleFreq (420dim)
RWC	73.3 ± 1.2 %	57.5 ± 0.9 %	93.5 ± 0.7 %	93.8 ± 0.8 %	97.7 ± 0.4 %	97.5 ± 0.5 %	98.5 ± 0.2 %
Notes	69.6 ± 1.9 %	67.0 ± 1.9 %	93.0 ± 0.9 %	90.0 ± 0.6 %	96.0 ± 0.8 %	96.1 ± 1.1 %	97.7 ± 0.5 %

**Table 5:** Regions of high mismatch between the RWC and solo datasets

	Rates (Hz)	Scales (c/o)	Frequency (kHz)
Piano	8–45	1.4–5.7	0.23–0.74
Violin	> 32	> 2	0.29–3.8
Cello	> 32	> 2	0.68–2.15
Saxophone	8–32	> 2	< 0.72
Clarinet	> 8	1–5.7	< 0.54
Flute	> 16	< 1	0.5–4.6

## Appendix

**Table 6:** List of sources in the solo music database

Album title	Year recorded	Instrument
Music for solo piano (1960–2001)	2007	Piano
Music for solo piano	2005	Piano
Six sonatas and partitas for violin solo	2006–7	Violin
Sequenza	1994	Violin
Bach cello suites	1982	Cello
Sonatas nos. 1 and 2 for unaccompanied cello	2009	Cello
Solo cello : 20th century works for solo cello	1999	Cello
Yamaon	1997	Saxophone
Exhibition of saxophone	2002	Saxophone
Absolute solo!	1996	Saxophone
Music for solo clarinet and orchestra	2001	Clarinet
Three pieces for clarinet solo	1986	Clarinet
Clarinet XX Vol1	2002	Clarinet
Michael Nyman Yamamoto Perpetuo for solo flute	2008	Flute
Cornucopia	2003	Flute
The hallelujah tree	2005	Flute
And blue sparks burn	2006	Flute
Summer Shimmers	2008	Flute
Gardens of Anna Maria Luisa de Medici	2005	Flute
